# Respiratory epithelial adenomatoid hamartoma of the anterior nasal septum a rare localisation of an unusual tumour in a child: a case report

**DOI:** 10.4076/1757-1626-2-8151

**Published:** 2009-09-16

**Authors:** Mariusz Gajda, Olaf Zagolski, Agnieszka Jasztal, Grzegorz J Lis, Grazyna Pyka-Fosciak, Jan A Litwin

**Affiliations:** 1 Department of Histology, Jagiellonian University, Medical CollegeKopernika 7, 31-034, KrakowPoland; 2 Department of ENT, Diagnostic and Therapeutic Medical Center “Medicina”Barska 12, 30-307 KrakowPoland

## Abstract

**Introduction:**

Hamartomas are non-neoplastic lesions constituted by a mixture of tissues indigenous to the region. Respiratory epithelial adenomatoid hamartomas are characterised by glandular proliferation lined by ciliated airway epithelium. Their localisation in the nasal cavity is rare and most frequent cases described so far were associated with the posterior nasal septum.

**Case presentation:**

A 9-year-old Caucasian boy presented with long-standing nasal obstruction. A large right nasal mass was evident on physical and CT examinations. It was surgically removed from the anterior nasal septum under general anaesthesia. Histologically, the diagnosis of REAH was established. The tumour lined by stratified squamous and ciliated respiratory epithelium was characterised by prominent glandular proliferation. By immunohistochemistry, the tumour was positive for cytokeratins, smooth muscle actin, vimentin, laminin, collagen type IV, CD8, and CD68. No S-100 immunoreactivity was observed. The patient has been asymptomatic for 12 months with completely healed lining of the nose.

**Conclusion:**

Respiratory epithelial adenomatoid hamartoma, although rare, must be taken into consideration in differential diagnosis of nasal exophytic lesions.

## Introduction

Glandular lesions of the sinonasal tract are uncommon and may cause a diagnostic dilemma. If common salivary gland tumours are excluded, most of the other glandular lesions include respiratory epithelial adenomatoid hamartomas (REAHs), inverted schneiderian papillomas and sinonasal adenocarcinomas (SNACs) of the intestinal (ITAC) and nonintestinal (non-ITAC) types [1]. Hamartomas are benign, non-neoplastic lesions constituted by a mixture of tissues which are indigenous to the region. They result from inborn errors of tissue development [2]. REAHs are characterised by glandular proliferation lined by ciliated epithelium originating from the airway epithelium [2]. Their localisation in the nasal cavity is unusual [3,4], with more common occurrence in males [5]. Association with nasal polyps supports the hypothesis that inflammation could be one of the inducing factors [4]. In the nasal cavity, REAHs are mostly associated with the posterior nasal septum [6], although lesion arising from the lateral nasal wall has also been reported [4]. The involvement of the maxillary sinus is extremely rare [6], although occasional occurrence of REAHs in areas surrounding nasal cavity, such as the ethmoid sinus, frontal sinus and nasopharynx has been described [5]. Apart from REAHs, folliculo-sebaceous cystic hamartomas, localised in the skin of the vestibule or around the nose [7], and sinonasal fibro-osseous hamartomas, originating from the bones of the nose and paranasal sinuses [8] have been reported in the sinonasal tract. Complete surgical resection of a REAH is the treatment of choice [4].

## Case presentation

**Figure 1.**
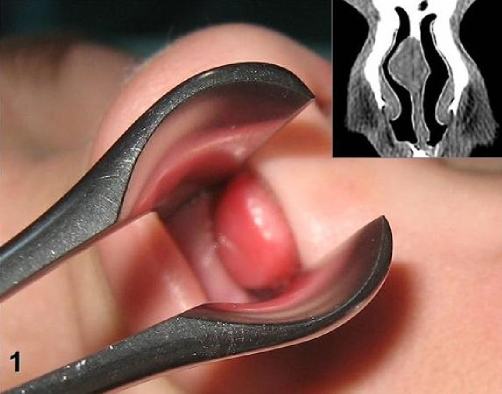
The tumour filling nearly the whole right nasal meatus of 9-year-old child and coronal view of preoperative CT image demonstrating right nasal mass adjacent to the nasal septum.

**Figure 2.**
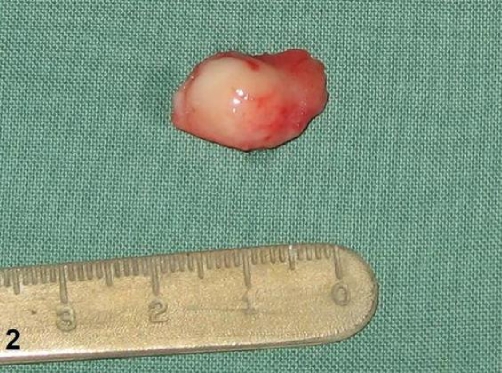
The tumour resected *en bloc*.

A 9-year-old Caucasian boy presented with long-standing nasal obstruction. Physical examination revealed a large mass covered with normal mucosa, originating from the right side of nasal septum and located 3 mm far from the vestibule. The lesion was polypoid in shape, soft to palpation but more indurate than inflammatory nasal polyp (Figure 1). CT scans confirmed a large right nasal mass adjacent to the nasal septum (Figure 1). The tumour was 15 × 10 × 10 mm in size and skin-to-pink coloured. It filled nearly all the right nasal meatus. Under general anaesthesia and endoscopic guidance, an incision posterior to the lesion was performed, the mass was separated from the quadrangle cartilage and dissected out with a 2 mm margin of unaffected mucosa (Figure 2). Since then, the patient has been asymptomatic for 12 months with completely healed lining of the nose.

**Figure 3.**
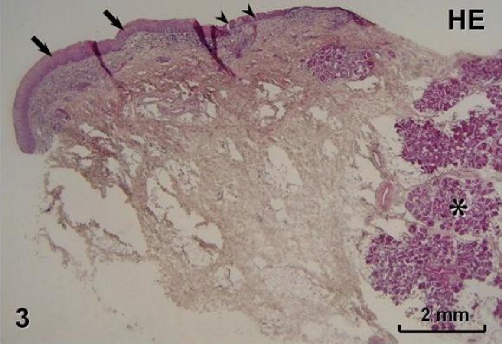
Fragment of the tumour showing transition between hypertrophied mucosa and glandular proliferation (asterisk) lined by stratified squamous (arrows) and ciliated respiratory epithelium (arrowheads), respectively. HE staining.

**Figure 4.**
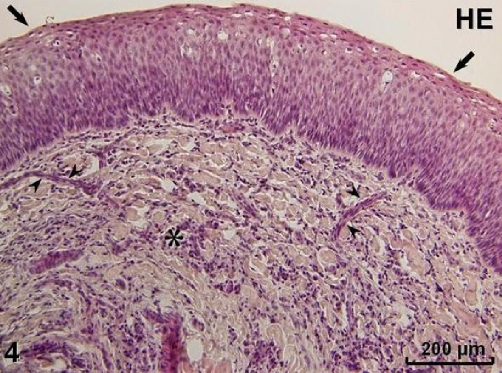
Parakeratinized stratified squamous epithelium (arrows) and inflammatory infiltration in connective tissue stroma (asterisk) with numerous blood vessels (arrowheads). HE staining.

Histopathologically (HE staining), prominent glandular proliferation containing serous as well as mucous components (Figure 3) was found in the tumour except for its anterior part where hypertrophied mucosa with prominent fibrous lamina propria without glands was observed. The latter portion of the lesion was lined by parakeratinized stratified squamous epithelium, whereas the glandular area was covered by ciliated respiratory epithelium (Figures 3, 4). In the connective tissue areas, abundant inflammatory cells and numerous blood vessels were present (Figure 4). No identifiable mitotic activity or necrosis could be observed.

**Figure 5.**
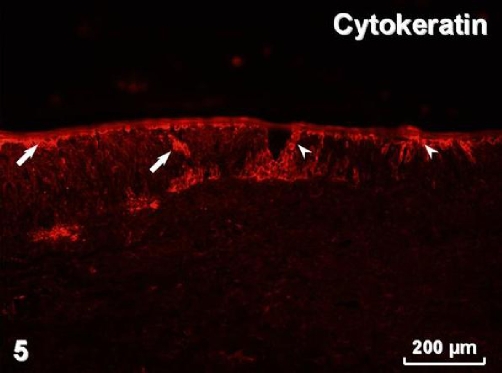
Pan-cytokeratin immunostaining in cells of the stratified squamous (arrows) and airway (arrowheads) epithelium.

**Figure 6.**
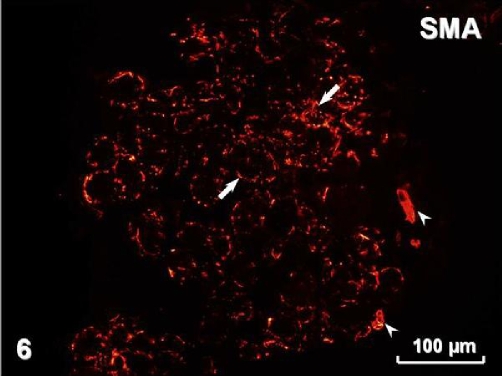
Smooth muscle actin immunoreactivity in vascular smooth muscle cells (arrowheads) and in myoepithelial cells (arrows).

**Figure 7.**
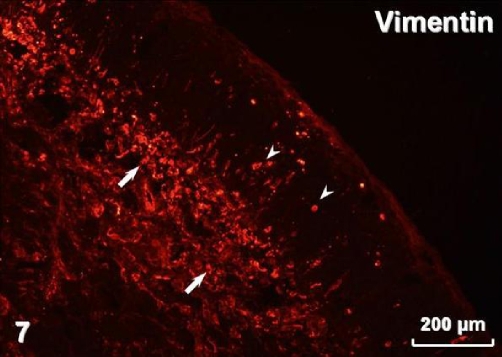
Vimentin immunoreactivity in cells of connective tissue stroma (arrows) and in the epithelium (arrowheads).

**Figure 8.**
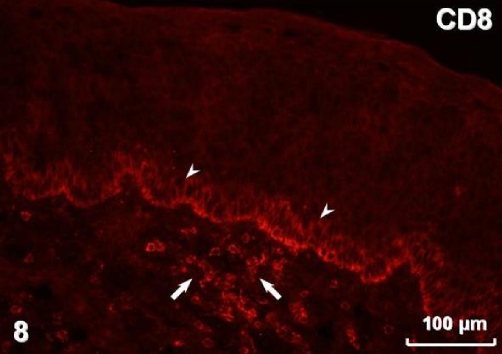
T lymphocytes in the stroma (arrows) visualised by CD8 immunostaining; note CD8-immunopositive cells in the basal layer of the epithelium (arrowheads).

**Figure 9.**
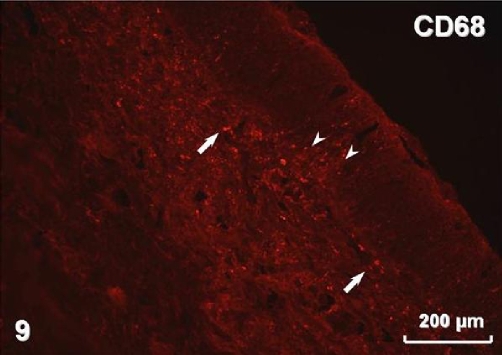
Macrophages in the stroma (arrows) and in the epithelium (arrowheads) visualised by CD68 immunostaining.

**Figure 10.**
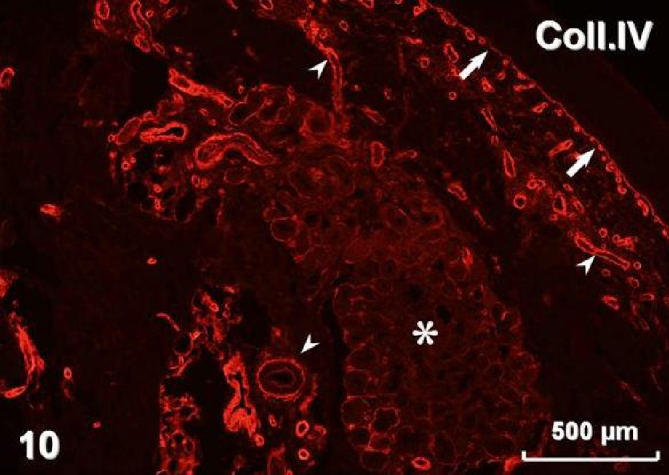
Collagen IV immunostaining in basal lamina of the epithelial lining (arrows), as well as in basal laminae of blood vessels (arrowheads) and glands (asterisk).

Immunohistochemistry revealed pan-cytokeratin (cytokeratins 4, 5, 6, 8, 10, 13, 18) immunostaining in both types of surface lining epithelium (Figure 5) as well as in glands. Smooth muscle actin was present in myoepithelial cells surrounding glandular acini and in blood vessel walls (Figure 6). Immunostaining for vimentin visualised positive cells located in the connective tissue and, occasionally, in the stratified and airway epithelium (Figure 7). Positive immunostaining for CD8 disclosed T lymphocytes in the stroma, but interestingly, cells of the basal layer of the stratified epithelium also presented CD8 immunoreactivity (Figure 8). Numerous macrophages expressing CD68 were observed in the connective tissue and, occasionally, in the epithelium (Figure 9). There was no S-100 immunoreactivity in the lesion (not shown). Collagen type IV (Figure 10) and laminin (not shown) were present in basal laminae of surface lining epithelium, blood vessels and glands.

## Discussion

The reported localisation of the tumour is unusual and REAHs have rarely been described in children [2,3]. The awareness of possible occurrence of this lesion in the discussed site is important for differential diagnosis. Inverted schneiderian papilloma and adenocarcinoma should always be considered in the sinonasal tract [4,6]. Despite characteristic histological features that distinguish these tumours, the differential diagnosis can be challenging in cases of small or fragmented endoscopic biopsies [1]. Appreciable allelic loss within REAH, observed in previous studies, supports the hypothesis that the lesion might be a benign neoplasm rather than a developmental malformation [9].

Ki-67 staining has been found restricted to the basal/myoepithelial layer, demonstrating a low proliferation index [1]. In our case there was also no visible mitotic activity. REAHs reported by Ozolek et al. [1] were positive for cytokeratin but negative for smooth muscle actin, S-100 protein, calponin, CK20, and CDX-2. They postulated that because of absence of myoepithelial markers, the cells surrounding secretory acini should be considered basal and not myoepithelial cells. In the lesion presented in this case, we observed myoepithelial cells expressing smooth muscle actin. Our REAH contained cells immunostained for vimentin in the stroma and also in the lining epithelia. Vimentin content is typical for connective tissue cells but it has also been found in normal respiratory epithelium [10]. However, it seems most likely that occasional intraepithelial vimentin-positive cells observed in our tumour represented migratory cells. The tumour was accompanied by chronic inflammation, which is in accordance with previous reports [4]. Positive reaction for CD8 disclosed numerous T lymphocytes, typically observed in chronic inflammation of the nasal mucosa [11]. We have also found numerous CD68-immunoreactive macrophages, presumably belonging to the population of antigen presenting cells [12]. Distribution of collagen type IV and laminin revealed by immunostaining in the tumour did not deviate from the pattern observed in normal nasal mucosa [13].

## Conclusion

Respiratory epithelial adenomatoid hamartoma, although rare, must be taken into consideration in differential diagnosis of nasal exophytic lesions. Hamartoma of the anterior nasal septum can be effectively removed with indications and extent of the resection like in benign tumours.

## Abbreviations

CT: computed tomography
HE: hematoxylin and eosin
ITAC: intestinal type adenocarcinoma
non-ITAC: non-intestinal type adenocarcinoma
REAH: respiratory epithelial adenomatoid hamartomas
SNAC: sinonasal adenocarcinoma

## References

[bib-001] Ozolek JA, Barnes EL, Hunt JL (2007). Basal/myoepithelial cells in chronic sinusitis, respiratory epithelial adenomatoid hamartoma, inverted papilloma, and intestinal type and nonintestinal-type sinonasal adenocarcinoma: an immunohistochemical study. Arch Pathol Lab Med.

[bib-002] Terris MH, Billman GF, Pransky SM (1993). Nasal hamartoma: case report and review of the literature. Int J Pediatr Otorhinolaryngol.

[bib-003] Picciotti PM, Calò L, Mulè A, Maggiore C, Scarano E (2008). Rhino sinusal bilateral hamartoma: A case report. Auris Nasus Larynx.

[bib-004] Delbrouck C, Fernandez Aguilar S, Choufani G, Hassid S (2004). Respiratory epithelial adenomatoid hamartoma associated with nasal polyposis. Am J Otolaryngol.

[bib-005] Wenig BM, Heffner DK (1995). Respiratory epithelial adenomatoid hamartomas of the sinonasal tract and nasopharynx: a clinicopathologic study of 31 cases. Ann Otol Rhinol Laryngol.

[bib-006] Kessler HP, Unterman B (2004). Respiratory epithelial adenomatoid hamartoma of the maxillary sinus presenting as a periapical radiolucency: a case report and review of the literature. Oral Surg Oral Med Oral Pathol Oral Radiol Endod.

[bib-007] Suarez-Penaranda JM, Vieites B, Ramirez-Santos A, Fernandez-Redondo V, Toribio J, Del Rio E, Forteza-Vila J (2009). Clinicopathological and immunohistochemical findings in a series of folliculosebaceous cystic hamartoma. J Cutan Pathol.

[bib-008] Boudewyns AN, van Dinther JJ, Colpaert CG (2006). Sinonasal fibro-osseous hamartoma: case presentation and differential diagnosis with other fibro-osseous lesions involving the paranasal sinuses. Eur Arch Otorhinolaryngol.

[bib-009] Ozolek JA, Hunt JL (2006). Tumor suppressor gene alterations in respiratory epithelial adenomatoid hamartoma (REAH): comparison to sinonasal adenocarcinoma and inflamed sinonasal mucosa. Am J Surg Pathol.

[bib-010] Kasper M, Stosiek P (1990). The expression of vimentin in epithelial cells from human nasal mucosa. Eur Arch Otorhinolaryngol.

[bib-011] Hao J, Pang YT, Wang DY (2006). Diffuse mucosal inflammation in nasal polyps and adjacent middle turbinate. Otolaryngol Head Neck Surg.

[bib-012] Jahnsen FL, Gran E, Haye R, Brandtzaeg P (2004). Human nasal mucosa contains antigen-presenting cells of strikingly different functional phenotypes. Am J Respir Cell Mol Biol.

[bib-013] Sanai A, Nagata H, Konno A (1999). Extensive interstitial collagen deposition on the basement membrane zone in allergic nasal mucosa. Acta Otolaryngol.

